# Social signalling as a framework for second-person neuroscience

**DOI:** 10.3758/s13423-022-02103-2

**Published:** 2022-06-01

**Authors:** Roser Cañigueral, Sujatha Krishnan-Barman, Antonia F. de C. Hamilton

**Affiliations:** 1 Institute of Cognitive Neuroscience, University College London, Alexandra House, 17 Queen Square, London, WC1N 3AZ UK; 2 Department of Clinical, Educational and Health Psychology, University College London, 26 Bedford Way, London, WC1H 0AP UK

**Keywords:** Second-person neuroscience, Social interaction, Social signal, Audience effects

## Abstract

Despite the recent increase in second-person neuroscience research, it is still hard to understand which neurocognitive mechanisms underlie real-time social behaviours. Here, we propose that social signalling can help us understand social interactions both at the single- and two-brain level in terms of social signal exchanges between senders and receivers. First, we show how subtle manipulations of being watched provide an important tool to dissect meaningful social signals. We then focus on how social signalling can help us build testable hypotheses for second-person neuroscience with the example of imitation and gaze behaviour. Finally, we suggest that linking neural activity to specific social signals will be key to fully understand the neurocognitive systems engaged during face-to-face interactions.

## Introduction

Interest in the neuroscience of social interactions has grown rapidly in the past decade. Influential opinion papers have called for a new “second-person neuroscience” and for the study of face-to-face dynamics (De Jaegher et al., 2010; Risko et al., 2016; Schilbach et al., 2013). Building on these, researchers have begun to develop paradigms where two or more people interact (Konvalinka et al., 2010; Sebanz et al., 2006) and where brain activity is captured using hyperscanning (Babiloni & Astolfi, 2014; Montague et al., 2002)*.* However, it is still not easy to pin down specific cognitive models of the processes engaged when people take part in dynamic, real-time interactions. That is, what kind of neurocognitive models can we use to make sense of dynamic social interactions?

Here, we propose that a social signalling framework can help us understand social interactions both at the single- and two-brain level in terms of signal exchanges between senders and receivers. Social signalling takes an incremental approach to this problem, asking what factors change between situations where one participant performs a task alone and the same situation where the participant is interacting with another person as they perform a task together. In particular, we suggest that the simple manipulation of “being watched” or not by another person provides a core test of social signalling and may be able to give us a robust and general theoretical framework in which to advance “second-person neuroscience.”

First, we briefly review evidence that “being watched” matters to participant’s behaviour and their brain activity patterns. We then outline the social signalling framework to understanding these changes, and we detail how this can be applied to understand two cases of social behaviour—imitation and eye gaze. Finally, we review emerging evidence on the neural mechanisms of social signalling and set out future directions.

## Being watched as a basic test of social interactions

There is a long tradition of research into the differences in our behaviour when we are alone, versus when we are in the presence of others. A series of studies from Zajonc (1965) showed that cockroaches, rats, monkeys, and humans showed changes in behaviour when in the presence of a conspecific, a phenomenon described as *social facilitation*. It has been proposed that the presence of conspecifics (regardless of whether they are watching) increases arousal and facilitates dominant behaviours, in both cognitive and motor tasks (Geen, 1985; Strauss, 2002; Zajonc & Sales, 1966).

In humans, the effect of being watched by another person goes beyond mere social facilitation and has been described as an *audience effect* (Hamilton & Lind, 2016). Being watched is one of the most basic and simplest social interactions, first studied by Triplett more than 100 years ago (Triplett, 1898), when he showed that children wind in a fishing reel faster when with another child than when alone. Since then, several studies have shown how an audience can induce the belief in being watched and cause changes in behaviour and in underlying brain activity. The audience effect is most clearly induced by the physical presence of another person who is actively watching the participant but can also be induced by the feeling of being watched (e.g., via camera), and these different triggering conditions are reviewed below. For instance, participants tend to gaze less at the face of a live confederate when compared with the same confederate in a prerecorded video clip (Cañigueral & Hamilton, 2019a; Gobel et al., 2015; Laidlaw et al., 2011), and they smile more in the presence of a live friend or confederate (Fridlund, 1991; Hietanen et al., 2018). During economic games and social dilemmas, the belief in being watched leads to an increase in prosocial behaviour (Cañigueral & Hamilton, 2019a; Izuma et al., 2009; Izuma et al., 2011) and a decrease in risk-taking (Kumano et al., 2021) as well as more brain activity in regions linked to mentalizing and social reward processing (Izuma et al., 2009, 2010).

**Fig. 1:**
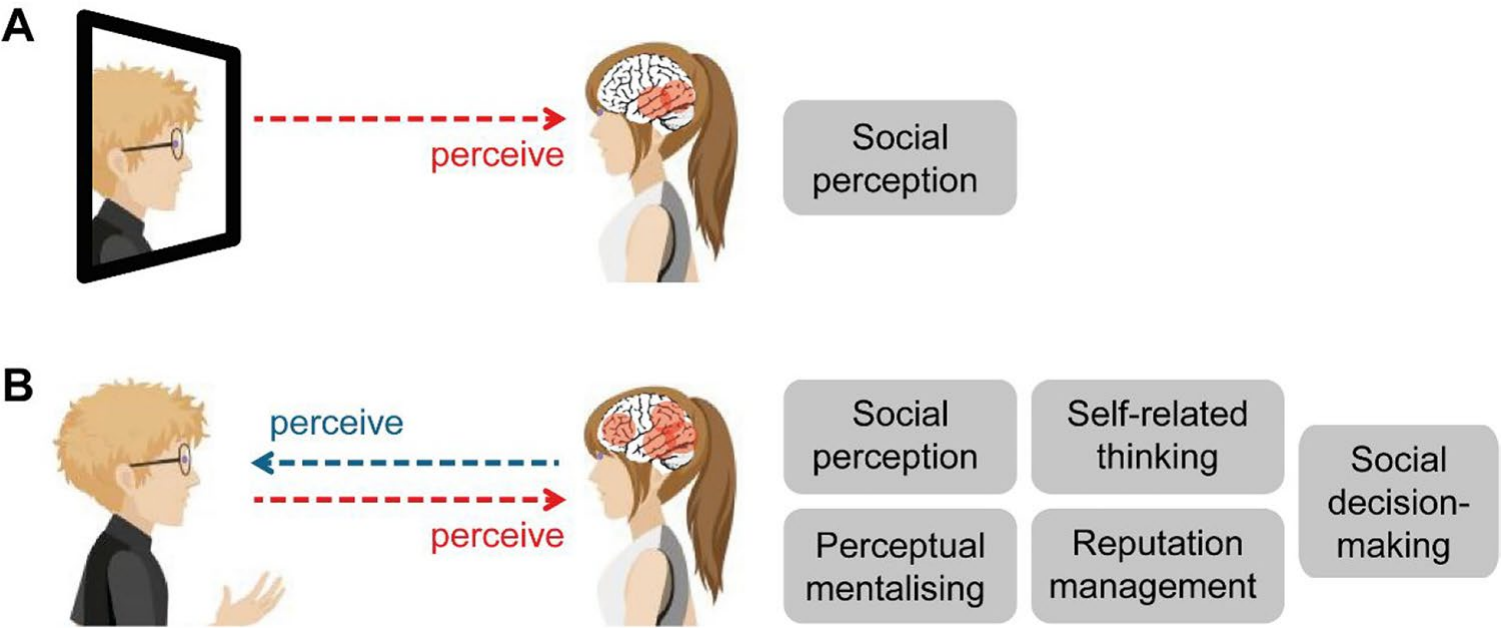
**a** Viewing a picture or movie of another person engages processes of social perception only. **b** Seeing another person face to face means that it is possible, and socially important, to consider what the other person can see. This may engage processes of perceptual mentalizing, self-referential thinking, reputation management and social decision-making

Several different cognitive mechanisms have been proposed to account for audience effects (Fig. 1). The response to being watched may draw on perceptual mentalizing (i.e., the attribution of perceptual states to other people; Teufel et al., 2010) to determine what the other person can *see*, and theory of mind (Tennie et al., 2010) to determine what they *think*. The presence of “watching eyes” could engage self-referential processing, which increases the sense of self-involvement in the interaction (Conty et al., 2016; Hazem et al., 2017). Furthermore, Bond (1982) proposed a self-presentation model of audience effects, whereby participants change their behaviour to present themselves positively to the audience. This also fits with the recent idea that being watched engages reputation management mechanisms—that is, changes in behaviour that aim to promote positive judgements in the presence of others (Cage, 2015; Izuma et al., 2009, 2010). However, there is still uncertainty about how to best interpret these findings and integrate them with other aspects of social neuroscience. Crucially, common to all these models is the idea that participants can send information about themselves to the watcher, that is, they can communicate.

## From being watched to social signalling

To make sense of these very basic types of communication, we believe that it is helpful to draw on the extensive studies on signalling in animal behaviour (Stegmann, 2013). In this tradition, a signal is defined as a stimulus which sends information from one individual to another, and which is performed in order to benefit both the sender and the receiver. In contrast, a cue is a stimulus which only benefits the receiver. For example, the carbon dioxide which I breathe out is a cue to a mosquito that wants a meal but does not benefit me. In contrast, the bright colour of a butterfly’s wings is a signal to birds to avoid eating the butterfly, which benefits the butterfly in helping it to avoid predation. The concept of signalling described here is very broad, applying to all types of stimuli from wing colour (defined by evolution) to particular behaviours (which only occur in particular contexts and might be learnt). Here, we take this general idea and apply it to the much more specific case of human nonverbal behaviour. In particular, we suggest that it is helpful to use the idea of signalling to define which human actions are used as signals and what those signals mean.

The social signalling framework proposes that if a human action is a social signal, it must meet two basic criteria. First, the sender must produce the action in order to influence the receiver. Second, the action must have a beneficial impact on the receiver. Importantly, to test the first criteria, we can vary the presence of an audience who can receive the signal. If an action is performed when the sender can be seen and is suppressed when the sender cannot be seen, we have evidence that the action is being used as a signal. To test the second criteria, we must evaluate how the receiver’s behaviour or mental state changes when they perceive the action, that is, do receivers change their attitude to the sender when they receive a signal? Note that receiving a signal should benefit the receiver in the sense that they have gained information about the social world, even if that information is negatively valenced (e.g., learning that another person is hostile). However, we acknowledge that there are circumstances in which receiving additional social information via a signal may have negative consequences for the receiver (e.g., if sender is lying and uses the signal to manipulate the receiver); such circumstances are beyond the scope of this paper.

Overall, the social signalling framework takes an incremental approach to understand social interactions in terms of signal exchanges between senders and receivers. To compare cognitive processes between a solo task (e.g., one participant responding to a computer) and a dynamic social interaction (e.g., two participants in conversation) is very complex. By studying the specific processes which change between a solo task and a solo task with an audience, we hope it will be possible to incrementally specify the different cognitive processes involved in different types of social behaviour.

Social signalling builds on the basic premise of second-person neuroscience, that engaging in social interactions involves additional neurocognitive processes and social dynamics compared to not being in an interaction (Redcay & Schilbach, 2019; Schilbach et al., 2013), and sets out a concrete framework to establish testable hypotheses in the context of two-person interactions. In particular, social signalling suggests that we need to study and understand the interactive behaviour of both performers (senders) and observers (receivers). By using the simple manipulation of being watched or not being watched (i.e., varying the presence of an audience/receiver), we can test which behaviours are used as signals and define what information content the signals might carry (Bacharach & Gambetta, 2001; Skyrms, 2010). In contrast, by varying the presence of a social signal, we can test which effects, if any, this signal has on the receiver.

## What counts as an audience?

**Fig. 2:**
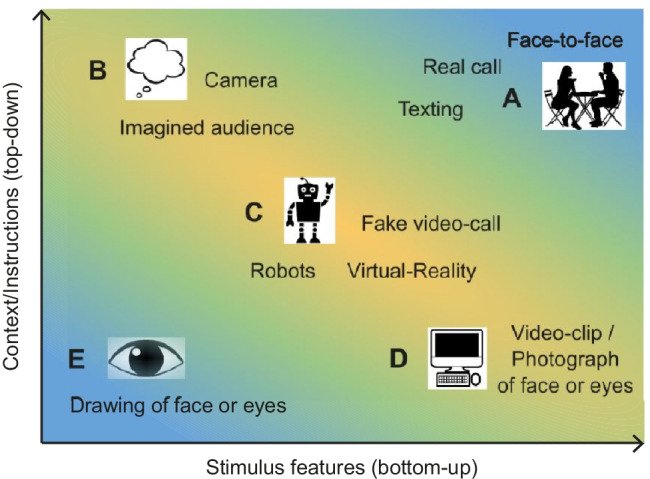
Common stimuli used in social neuroscience research represented in a 2D space, along a spectrum of top-down (*y*-axis) and bottom-up (*x*-axis) features. Studies in the top-right corner of the plot clearly involve “being watched” (**a**) while those in the bottom-left do not (**e**). Studies in the yellow zone may be more ambiguous (**b–d**). (Colour figure online)

To build a comprehensive account of social signalling, it is important to consider which manipulations count as being watched or not, and thus which engage the additional cognitive processes involved in audience effects. The extreme cases are most clear cut—when a participant is engaged in a face-to-face conversation with another living person, it is clear that they are being watched, while a participant who views a cartoon of a pair of eyes on a computer alone in a room (with no cameras) might see a face-like image but is not being watched. We illustrate these examples in Fig. 2, where we divide the space of possible interactions in terms of the visible stimulus features (e.g., an image of eyes) on the *x*-axis, and top-down contextual knowledge on the *y*-axis. The face-to-face conversation includes both visual cues to another person and the knowledge they are real and are watching (Fig. 2a) so an audience effect should be active. Instead, the person viewing cartoon eyes has minimal visual cues together with the knowledge they are not being watched (Fig. 2e), so the audience effect is not active.

However, there are many other possible contexts which are much more ambiguous, and where we might not know if an audience effect is present or not; these are illustrated on the yellow diagonal (Fig. 2b–d). These include cases where a participant sees a camera and is told “*you are being watched”* but sees no visual cues (Fig. 2b; e.g., Somerville et al., 2013). In other cases, a participant might see an image or video clip of a person who directly addresses them (Fig. 2d), which include rich visual cues but participants still know that this image or video clip cannot actually see them (e.g., Baltazar et al., 2014; Wang & Hamilton, 2012). Finally, a number of studies use a combination of instructions and visual stimuli to induce the belief that participants are or are not engaged in a social interaction, using fake video calls (Cañigueral & Hamilton, 2019a), fake mirrors (Hietanen et al., 2019), or virtual characters (Wilms et al., 2010; Fig. 2c).

In each of these cases, either the perceptual features of the stimulus or the instructions given by the experimenter may lead participants to feel as if they are being watched. In interpreting such studies, we make the assumption that the feeling of “I am watched by a person” is a categorical percept (Hari & Puce, 2010)—that is, in each case the participant either does feel watched or does not, with no feeling of being half watched. However, it is not easy to make a blanket rule for which stimuli in the “ambiguous zone” will be treated as “true watchers” by participants and which will not—subtle effects of context can have a large impact. Equally, it is possible that some participants interpret a particular context as “being watched” while other participants in the same study do not. Future studies that aim to define more clearly when an ambiguous stimulus is treated as an audience will be useful in understanding basic mechanisms involved in detecting humans and triggering audience effect processes.

In the following, we illustrate how a framework of social signalling can help us build testable hypotheses for second-person neuroscience by separately manipulating both the sender and the receiver in a two-person interaction. We particularly focus on the examples of imitation and gaze behaviour, although similar studies have examined other social behaviours including facial expressions (Crivelli & Fridlund, 2018), eye blinks (Hömke et al., 2017, 2018), and hand gestures (Holler et al., 2018; Mol et al., 2011).

## Imitation as an example of social signalling

Imitation is a simple social behaviour in which the actions of one person match the actions of the other (Heyes, 2011). It is relatively easy to recognise (Thorndike, 1898; Whiten & Ham, 1992) but there are many theories of why people imitate (see Farmer et al., 2018, for a review). These include imitation to learn new skills (Flynn & Smith, 2012); imitation to improve our understanding of another person via simulation (Gallese & Goldman, 1998; Pickering & Garrod, 2013); imitation to affiliate with others (Over & Carpenter, 2013; Uzgiris, 1981); and imitation as a side effect of associative learning (Heyes, 2017). Here, we focus on the claim that imitation is used as a social signal in order to build affiliation with others, sometimes described as the social glue hypothesis (Dijksterhuis, 2005; Lakin et al., 2003; Wang & Hamilton, 2012). Note that other social signals need not be linked to affiliation, such as those behaviours aimed at signalling dominance or status (Burgoon & Dunbar, 2006).

**Fig. 3:**
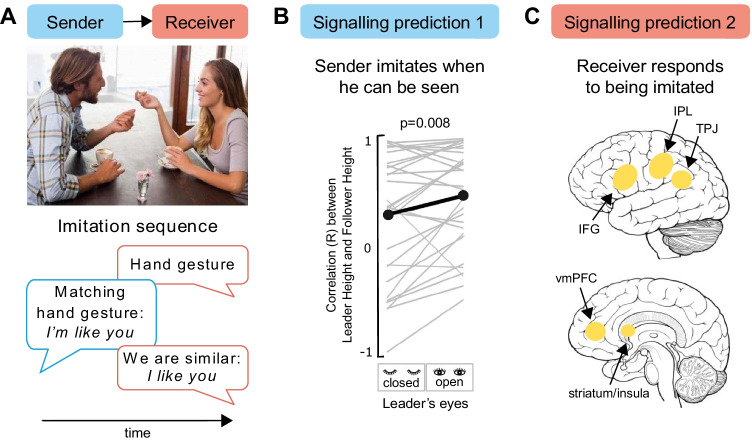
The social signalling framework in the context of imitation. In a typical imitation sequence (**a**), one person acts, the second copies, and the first responds to being imitated; this is represented as a pseudo-conversation, where red bubbles are the woman’s action/cognition and blue bubbles are the man’s action/cognition. Two predictions must be true to classify imitation as a signal. First, the imitator (sender) produces the action more when he can be seen (**b**); data in Panel **b** confirms this (Krishnan-Barman & Hamilton, 2019). Second, the imitatee (receiver) must detect on some level that she is being copied and must respond (**c**); brain systems linked to this process are summarised in Panel **c** (IPL = inferior parietal lobule; TPJ = temporo-parietal junction; IFG = inferior frontal gyrus; vmPFC = ventromedial prefrontal cortex; Hale & Hamilton, 2016a). (Colour figure online)

The claim that imitation acts as a social glue assumes that two people engage in an imitation sequence as shown in Fig. 3a. Here, the woman performs a hand gesture and the man then imitates her action: this is represented in the figure below as a pseudo-conversation, where red bubbles are the woman’s action/cognition and blue bubbles are the man’s action/cognition. When the man imitates the woman, his action is *sending* a signal to her. When she senses (probably implicitly) that she is being imitated, she *receives* the signal and may adjust her evaluation to like him more. If this interpretation of the imitation sequence under the social signalling framework is true, we can set out two testable hypotheses. First, imitation should be produced when other people can see it (Fig. 3b), because there is no need to send a signal if the signal cannot be received. Second, being imitated should change the internal state or behaviour of the receiver, as the receiver has gained new information about the sender (Fig. 3c).

In a study of dyadic interaction, we found support for the first prediction of the social signalling framework of imitation (Krishnan-Barman & Hamilton, 2019). Pairs of naïve participants in an augmented reality space were assigned roles of leader and follower in a cooperative game. On each trial, the leader learnt a block-moving sequence from the computer and demonstrated it to the follower, who was instructed to move the blocks in the same order; dyads received a score based on fast and accurate performance. Unbeknownst to the Follower, the Leader was given a secret instruction to move blocks using specific trajectories, including unusually high trajectories in some trials. In one half of the trials the Leader watched the Follower make their subsequent movements, while in the other half of the trials the Leader had their eyes closed during the Follower’s turn. We found that overall Followers tended to imitate the trajectories demonstrated by the Leaders, and critically, they imitated Leaders with greater fidelity when they knew they were being watched by the Leader (Fig. 3a).

The finding that being watched increases imitation is also seen in other contexts. A previous study (Bavelas et al., 1986) showed that observers winced more when watching an experimenter who was maintaining eye contact with them sustain a minor injury when compared with an experimenter who was looking elsewhere when experiencing a minor injury. Both toddlers (Vivanti & Dissanayake, 2014) and 4-month-old infants (de Klerk et al., 2018) show a greater propensity to imitate models in a video demonstration when cued with a direct rather than averted gaze. In a rapid reaction time study with adults, direct gaze enhances mimicry (Wang et al., 2011) and this effect is only seen if the gaze is present during the response period (Wang & Hamilton, 2014). However, in studies using direct gaze cues, it is hard to rule our arousal or alerting effects arising from the eyes (Senju & Johnson, 2009). Our recent paper (Krishnan-Barman & Hamilton, 2019) manipulated only the belief in being watched because participants stood side by side and did not directly see each other’s eyes. This suggests that the effect is not merely an epiphenomenon of arousal (Senju & Johnson, 2009) but is driven by the capacity to signal to the partner when the partner’s eyes are open. Together, all these results offer support for the hypothesis that imitation is a social signal initiated by the sender.

We now turn to the second hypothesis: if imitation is a social signal, then being imitated should (on some level) be detected by the receiver and this new information should change the internal state or behaviour of the receiver. Several detailed reviews outline the downstream impacts of being mimicked (Chartrand & Lakin, 2012; Chartrand & van Baaren, 2009; Hale & Hamilton, 2016a; but see Hale & Hamilton, 2016b). Broadly being mimicked appears to build rapport and increase our liking for other people (Chartrand & Bargh, 1999; Lakin & Chartrand, 2003; Stel & Vonk, 2010), and this effect is present from early in childhood (Meltzoff, 2007). Interestingly, this effect may persist even when the mimicker is a computer or virtual-reality agent (Bailenson & Yee, 2005; Suzuki et al., 2003; but see Hale & Hamilton, 2016b). In addition to building rapport, mimicking has also been shown to increase prosocial behaviour such as helping others (Müller et al., 2012) or increasing the tips that restaurant patrons give waitresses (van Baaren et al., 2003). Thus, positive behavioural consequences of being imitated seem well-documented, though the precise neural and cognitive mechanisms which allow us to detect “being mimicked” are less well defined (Hale & Hamilton, 2016a). Taken together, these results support the hypothesis that imitation is a signal, produced by a sender when they are being watched, and resulting in changes in behaviour among the recipients of this signal.

One important question in a signalling account of imitation concerns the level of intentionality and awareness of the signals, in both the sender and the receiver. Like many nonverbal behaviours, people often seem to be unaware of when they imitate others and of when others imitate them. In fact, awareness of being imitated may reduce the social glue effect (Kulesza et al., 2016). Thus, the social signalling framework makes no claims that people consciously intend to send a signal or are explicitly aware of receiving a signal, and it is possible that all these sophisticated processes can occur without awareness, in the same way that a tennis player can hit a ball without awareness of their patterns of muscle activity. It will be an interesting question for future studies to explore how intentions and awareness interact with nonverbal social signalling behaviours.

## Gaze as an example of social signalling

Gaze and eye movements are a particularly intriguing social behaviour, because the eyes are used to gather information about the world but can also signal information to others (Gobel et al., 2015). There is evidence that people change their gaze behaviour when they are being watched, which supports the first prediction of the social signalling framework. For instance, participants direct less gaze to the face of a live confederate than to the face of the same confederate in a prerecorded video clip (Laidlaw et al., 2011). Similarly, across two studies we recently showed that, when participants are in a live interaction or when they are (or believe they are) in a live video call, they gaze less to the other person than if they are seeing a prerecorded video clip (Cañigueral & Hamilton, 2019a; Cañigueral, Ward, et al., 2021).

Some studies have suggested that gaze avoidance found in live contexts signals compliance with social norms (e.g., it is not polite to stare at someone; Foulsham et al., 2011; Gobel et al., 2015; Goffman, 1963) or reduces arousal associated with eye contact in live interactions (Argyle & Dean, 1965; Kendon, 1967). However, other studies using tasks that involve conversation have shown that the amount of gaze directed to the confederate is greater when they are listening compared to speaking (Cañigueral, Ward, et al., 2021; Freeth et al., 2013). Moreover, one study found that in contexts involving natural conversation participants direct more gaze to the confederate when they believe they are in a live video call (Mansour & Kuhn, 2019). Altogether these findings suggests that, beyond being in a live interaction, the communicative context and role in the interaction also modulates gaze patterns (Cañigueral & Hamilton, 2019b).

In line with the second prediction of the social signalling framework, some studies show that live direct or averted gaze has effects on the receiver. Studies using pictures and virtual agents have shown that direct gaze can engage brain systems linked to reward (Georgescu et al., 2013; Kampe et al., 2001), but can also be processed as a threat stimulus (Sato et al., 2004). In live contexts, seeing direct or averted gaze activates the approach or avoidance motivational brain system, respectively (Hietanen et al., 2008; Pönkänen et al., 2011). In conversation, eye gaze regulates turn-taking between speakers and listeners: speakers avert their gaze when they start to speak and when they hesitate to indicate that they want to say something but give direct gaze to the listener when they are finishing an utterance to indicate that they want to give the turn (Ho et al., 2015; Kendon, 1967). Thus, the key role of eye gaze as a social signal emerges from its dual function as a cue of “being watched” and as a dynamic modulator of social interactions on a moment-by-moment basis (Cañigueral & Hamilton, 2019b).

## Neural mechanisms for social signalling

Within the social signalling framework, the exchange of social signals will also modulate brain mechanisms engaged by senders and receivers. At the sender’s end, brain activity should change depending on the presence or absence of an audience, and several studies have used creative paradigms to test this hypothesis inside the fMRI scanner. For instance, using mirrors it has been shown that mutual eye contact with a live partner recruits the medial prefrontal cortex, a brain area involved in mentalizing and communication (Cavallo et al., 2015). Using a fake video-call paradigm, it has also been shown that the belief in being watched during a prosocial decision-making task recruits brain regions associated with mentalizing (medial prefrontal cortex) and reward processing (ventral striatum), which are two key processes for reputation management (Izuma et al., 2009, 2010). Similarly, the belief in being watched or that an audio feed is presented in real-time (versus prerecorded) engages mentalizing brain regions (Müller-Pinzler et al., 2016; Redcay et al., 2010; Somerville et al., 2013; Warnell et al., 2018), and the belief of chatting online with another human (versus a computer) engages reward processing areas (Redcay et al., 2010; Warnell et al., 2018). These studies all point to the idea that a particular network of brain regions previously linked to mentalizing and reward are also engaged when a participant feels they can be seen by or can communicate with another person.

Commenting on patterns of brain activity related to receiving social signals from other people might seem simple, in the sense that hundreds of studies have itemized brain regions of social perception. Such studies do not typically distinguish whether a particular behaviour was intended as a signal or not but have identified brain systems which respond to emotional faces, to gestures, to actions and to observing gaze patterns (Andric & Small, 2012; Bhat et al., 2017; Diano et al., 2017; Pelphrey et al., 2004). When the finer distinction between a signal and a cue matters, it is helpful to consider studies which distinguish between ostensive and nonostensive behaviours. These have shown that ostensive communicative cues such as direct gaze, being offered an object, or hearing one’s own name recruit brain areas related to processing of communicative intent, mental states, and reward (Caruana et al., 2015; Kampe et al., 2003; Redcay et al., 2016; Schilbach et al., 2006; Schilbach et al., 2010; Tylén et al., 2012).

During social signalling, receivers also need to infer the intended message or “speaker meaning” embedded in a signal, which is strongly dependent on contextual information beyond the signal itself (e.g., based on assumptions about the senders’ beliefs and intentions; Hagoort, 2019). For instance, sustained direct gaze between participants interacting face-to-face can result in either laughter or hostility, according to the context set by a preceding cooperative or competitive task (Jarick & Kingstone, 2015). Thus, at the receiver’s end social signalling may also recruit brain systems involved in inferring such “speaker meaning.” Studies within the field of neuropragmatics have investigated this question in the context of spoken language, and have found that listening to irony, implicit answers or indirect evaluations and requests recruits the medial prefrontal cortex and temporal-parietal junction (Bašnáková et al., 2014; Jang et al., 2013; Spotorno et al., 2012; van Ackeren et al., 2012). Moreover, when participants are the receivers of the indirect message, versus just overhearers, listening to indirect replies also recruits the anterior insula and pregenual anterior cingulate cortex (Bašnáková et al., 2015). These findings suggest that mentalizing and affective brain systems are required to understand the “speaker meaning” and communicative intent of a signal (Hagoort, 2019; Hagoort & Levinson, 2014).

Although the studies presented above have advanced our understanding of how the brain implements a variety of cognitive processes when being watched or when receiving a social signal, they rely on controlled laboratory settings that require participants to be alone and stay still inside the fMRI scanner. This limits the researcher’s ability to study brain systems recruited in social interactions, where participants naturally move their face, head, and body to communicate with others. Luckily, these limitations can be overcome by techniques that allow much higher mobility (Czeszumski et al., 2020). For instance, although EEG has traditionally been highly sensitive to motion artifacts, recent developments have created robust mobile EEG (MEG) systems that can be easily used in naturalistic settings (Melnik et al., 2017). To a lesser extent, MEG has also successfully been used for studies involving natural conversation (Mandel et al., 2016). Finally, functional near-infrared spectroscopy (fNIRS) is a novel neuroimaging technique that can record haemodynamic signals in the brain during face-to-face interactions (Pinti et al., 2018). Crucially, the fact that EEG and fNIRS are silent and wearable means that they can be easily combined with other methodologies that capture natural social behaviours, such as motion capture (mocap), face-tracking and eye-tracking systems.

For instance, by combining mocap and fNIRS to study imitation in a dyadic task, we have recently found that when participants are being watched as they perform an imitation task, there was a decrease in activation of the right parietal region and the right temporal-parietal junction (Krishnan-Barman, 2021). In another study (Cañigueral, Zhang, et al., 2021), we simultaneously recorded pairs of participants (who were facing each other) with eye-tracking, face-tracking, and fNIRS to test how social behaviours and brain activity are modulated when sharing biographical information. Results showed that reciprocal interactions where information was shared recruited brain regions previously linked to reputation management (Izuma, 2012), particularly to mentalizing (temporo-parietal junction; [TPJ]) and strategic decision-making (dorsolateral prefrontal cortex [dlPFC]; Saxe & Kanwisher, 2003; Saxe & Wexler, 2005; Soutschek et al., 2015; Speitel et al., 2019).

Within the social signalling framework, it is necessary to link brain activity patterns to meaningful social signals to fully understand the neurocognitive systems engaged during social interactions. In an exploratory analysis, we investigated how the amount of facial displays is related to brain activity in face-to-face interactions (Cañigueral, Zhang, et al., 2021). We found that spontaneous production of facial displays (i.e., participants moving their own face) recruited the left supramarginal gyrus, whereas spontaneous observation of facial displays (i.e., participants seeing their partner move the face) recruited the right dlPFC. These brain regions have been previously linked to speech actions (Wildgruber et al., 1996) and emotion inference from faces (A. Nakamura et al., 2014; K. Nakamura et al., 1999), respectively. However, these findings also suggest that these brain regions are able to track facial displays over time (as the interaction develops), and further reveal that there may be specific brain systems involved in the dynamic processing of social signals beyond those traditionally linked to motor control and face perception.

Other studies have taken advantage of EEG and fNIRS to study how two brains synchronize when two people are interacting face-to-face and exchange specific social signals. For instance, dual brain and video recordings of hand movements show that cross-brain synchrony increases during spontaneous imitation of hand movements (Dumas et al., 2010). In combination with eye-tracking systems, it has also been shown that cross-brain synchrony between partners increases during moments of mutual eye contact (Hirsch et al., 2017; Leong et al., 2017; Piazza et al., 2020). To further test which specific aspects of social signalling are modulated by cross-brain synchrony, we combined behavioural and neural dyadic recordings with a novel analytical approach that carefully controls for task- and behaviour-related effects (cross-brain GLM; Kingsbury et al., 2019; Cañigueral, Zhang, et al., 2021). We found that, after controlling for task structure and social behaviours, cross-brain synchrony between mentalizing (right TPJ) and strategic decision-making regions (left dlPFC) increased when participants were sharing information. In line with the mutual prediction theory (Hamilton, 2021; Kingsbury et al., 2019), this finding suggests that cross-brain synchrony allows us to appropriately anticipate and react to each other’s social signals in the context of an ongoing shared interaction.

Altogether, these studies demonstrate how a multimodal approach to social interactions is crucial to fully understand the neurocognitive systems underlying social signalling. Simple manipulations of the belief in being watched or communicative context show that mentalizing, reward and decision-making brain systems are engaged when participants take part in a live interaction. Beyond this, specific brain regions related to speech production and emotion processing track the dynamic exchange of social signals, while cross-brain synchrony might index the participants’ ability to anticipate and react to these signals. Future studies that carefully manipulate the social context and combine novel technologies to capture both brain activity and social behaviours will be critical to discern the role of each of these mechanisms in social signalling, as well as how they are all coordinated to enable real-world face-to-face social interactions.

## Taking the social signalling framework further

The ideas about social signalling outlined here provide a very minimal version of this framework. We suggest that the simple manipulation of “being watched” or not by another person provides a core test of social signalling, and that the two key features required to identify a signal are that the sender intends (on some level) to send a signal and that the receiver reacts (in some way) to the signal. Researchers in animal communication often examine further criteria. For example, persistence by the sender provides evidence that a signal is important—if the sender does not see any reaction from the recipient and the signal matters, the sender will keep sending it. Such behaviour implies that the sender has a goal of “she must get the message” and will persist until the goal is achieved. Other studies of animal behaviour consider if a particular signal is honest or deceptive (Dawkins & Guilford, 1991), and how recipients can distinguish these. Although our basic framework does not include these additional features, testing for them could be useful to have a more complex and detailed approach to social signalling.

Similarly, it is important to consider how social signalling might be modulated along different dimensions. For instance, while in many situations social signals are overt, in some cases there will be *covert* social signalling to facilitate effective cooperation: covert signals can be accurately received by its intended audience to foster affiliation, but not by others if it may lead to dislike (Smaldino et al., 2018). From the point of view of the audience, social signalling also differs in its directedness, that is, signals can be directed to “me,” or to a third person, or can also be undirected (e.g., face-covering tattoos; Gambetta, 2009). Importantly, depending on their directedness social signals will recruit different brain systems (Tylén et al., 2012). Another intriguing aspect is the context in which social signalling takes place, and how we adapt (or not) social signals to each of these contexts. For example, nonverbal behaviours such as nodding or hand movements play a central role in face-to-face communication to convey the full complexity of a spoken message (Kendon, 1967, 1970), but we continue to perform them in a telephone conversation although they can no longer be seen by the receiver. Thus, social signalling should allow for the nuance present in real-world contexts when testing which behaviours count or not as social signals. Finally, inspired by fields like conversation analysis (Schegloff, 2007), the investigation of social interactions within a social signalling framework entails considering signals as components within sequences of interaction instead of isolated entities, where prior and subsequent signals determine the relevance and meaning of the current signal. Acknowledging these dimensions when designing and interpreting studies will be critical to avoid an oversimplified view of social signalling.

It is also helpful to consider how this signalling framework relates to other approaches to the study of social interaction, and we briefly describe two rival approaches. First, some have suggested that we should focus entirely on the interaction and the emergent features of that situation (De Jaegher et al., 2010). Such dynamical systems often eschew traditional descriptions of single brain cognition, and of individuals as “senders” and “receivers.” There may well be situations, such as understanding the dynamic coordination of pianists playing a duet, where a division of the interaction into two distinct roles does not help. However, as the examples above illustrate, there are many situations where it is useful to understand who is sending a signal, what signal that is and how the recipient responds.

Second, some theories of social behaviour draw on studies of linguistic communication to interpret actions in terms of many different levels of relevance (Sperber & Wilson, 1995), where each action is tailored to the needs of the recipient. Such careful and detailed communication may be found for verbal behaviour, but it is not clear that the same models can be imported as a framework for all types of social interaction. Our approach here deliberately draws on work from animal cognition, which makes minimal assumptions about the complexity of the cognitive processes underlying social signalling. A key challenge is to understand if signalling can be driven by simple rules or if it requires the full complexity of linguistic communication.

In the present paper, we aim to highlight a “mid-level” type of explanation as a useful framework for interpreting current studies and guiding future studies. We suggest that a social signalling framework gives us a way to understand two-person interactions at the single-brain level, in the context of all our existing cognitive neuroscience. We aim to define precisely what signals each individual sends and receives during an interaction and the cognitive processes involved. These cognitive processes happen within a single brain but only in the context of a dynamic interaction with another person.

## Concluding remarks

Recent calls for second-person neuroscience have resulted in a significant body of research focused on two-person interactions. However, it is not yet clear which neurocognitive mechanisms underlie these real-time dynamic social behaviours, or how novel interactive methods can relate to findings from traditional single-brain studies of social cognition. The social signalling framework proposes that communication is embodied in social behaviours, and so must be instantiated in the physical world via signals embedded in motor actions, eye gaze or facial expressions. We propose that a social signalling framework can help us make sense of face-to-face interactions by taking step-by-step advances from traditional one-person studies to novel two-person paradigms (e.g., subtle manipulations of being watched). Key to this work is to understand the details of the signals—to identify a specific signal, link it to a context, understand when it is produced, and understand what effect it has on the receiver. We believe that, without a detailed understanding of signalling behaviours, it will be hard to make sense of the new wave of data emerging from second-person neuroscience methods.

## Data Availability

Not applicable.
